# The Ethical Use of Fit Indices in Structural Equation Modeling: Recommendations for Psychologists

**DOI:** 10.3389/fpsyg.2021.783226

**Published:** 2021-11-23

**Authors:** Bryant M. Stone

**Affiliations:** Department of Psychology, Southern Illinois University Carbondale, Carbondale, IL, United States

**Keywords:** structural equation modeling, factor analysis, ethical issues, model fit, fit indices

## Abstract

Fit indices provide helpful information for researchers to assess the fit of their structural equation models to their data. However, like many statistics and methods, researchers can misuse fit indices, which suggest the potential for questionable research practices that might arise during the analytic and interpretative processes. In the current paper, the author highlights two critical ethical dilemmas regarding the use of fit indices, which are (1) the selective reporting of fit indices and (2) using fit indices to justify poorly-fitting models. The author highlights the dilemmas and provides potential solutions for researchers and journals to follow to reduce these questionable research practices.

## Introduction

Structural equation modeling (SEM) allows researchers to analyze data in ways that are impossible under the general linear model, such as simultaneously assessing multiple relationships across variables or measuring variables that researchers cannot directly observe (i.e., latent variables) such as depression or self-esteem. Many modern scales and measures within the social sciences and education, such as intelligence tests, personality assessments, and diagnostic tools for mental health professionals, use structural equation modeling to align measures with underlying latent constructs. Researchers must create a model, collect the data, and then test the model’s fit to the collected data. Although there are many ways to assess for model fit, many researchers rely on fit indices, a collection of statistics that quantify the degree of data-model fit. These measures may assist researchers in judging the fit of their models. However, like many statics and methods, researchers may misuse fit indices through unethical and questionable research practices. The current paper investigates and suggests future directions for two ethical dilemmas regarding fit indices: the selective reporting of fit indices to bias the apparent fit of a model and the use of fit indices to justify poorly fitted models.

## Fit Indices

Researchers have categorized the dozens of fit indices into four broad domains (Hu and Bentler, 1999). First, researchers calculate absolute fit indices (e.g., standardized root-mean-square residual) by comparing the observed covariance matrix (i.e., the collected data) to the implied covariance matrix (i.e., the covariances that arose from the specified model). Second, relative fit indices (e.g., Tucker-Lewis Index) compare the specified model to a baseline model. A baseline model is a model where all the observed variables or collected data are uncorrelated. Third, noncentrality-based indices (e.g., Comparative Fit Index or the Root-Mean-Square Error of Approximation) are indices that adjust the perfect fit of the model, so that the chi-square equals the model’s degrees of freedom instead of zero. Fourth, parsimonious fit indices (e.g., Parsimonious Goodness of Fit Index) tend to be fit indices from other categories adjusted to favor more parsimonious models over more complex models. These fit indices quantify the model fit through multiple methods.

## The Selective Reporting of Fit Indices

Fit indices are easily biased and demonstrate considerable variability. Some fit indices are less vulnerable to the influence of extraneous variables, such as the CFI and RMSEA (Cangur and Ercan, 2015). However, some estimation techniques significantly inflate the standardized root mean squared residual (SRMR; e.g., generalized least squares technique is inflated compared to the asymptotically distribution-free technique). Other studies have found that the sample size easily biases the Tucker-Lewis index (TLI) and the normed fit index (NFI; Yadama and Pandey, 1995). Further, some fit indices, such as the CFI, TLI, and RMSEA, are biased to favor bifactor models (Morgan et al. 2015). The varying sensitivity to extraneous factors increases the amount of variability across fit indices.

The significant variability across fit indices may influence researchers to report those indices that suggest the best model fit. Many SEM software packages (e.g., *R* or LISREL) automatically calculate multiple fit indices when performing the initial SEM analyses. The automatic calculation of multiple fit indices allows researchers to observe and report the fit indices that support their model’s fit. For example, individuals with more complicated models may choose not to report parsimonious fit indices, which favor simpler models; and individuals with larger sample sizes may choose to report the NFI or NNFI, which favor models when sample sizes are large. This selective reporting may mislead the readers to believe the specified model fits the data better than it does. Therefore, fit indices provide a wide range of useful information about the data-model fit; however, researchers may engage in questionable research practices by selectively reporting certain fit indices.

## Future Directions

Two potential solutions may limit researchers’ ability to selectively report fit indices that justify their model. First, journals may consider standardizing the fit indices that they publish in their journal. Journals tend to have few standards for publishing SEM analyses, particularly fit indices. For example, a sample of 194 papers published by the American Psychological Association found that over 75% of articles that contain confirmatory factor analyses report the CFI and RMSEA (Jackson et al. 2009). Still, there was significant variability with the reported fit indices, with 34% reporting the Goodness of Fit Index (GFI), 23% reporting the NFI, and 46% reporting the TLI. Thus, the evidence suggests that journals may need more standardization of fit index reporting. In addition, journals have a responsibility to prevent the publication of articles created using unethical research practices. Still, some might argue that it is not the journal’s responsibility to assure that their articles follow a standard of reporting fit indices. Instead, some might argue that it is the reviewers’ responsibility to assure proper reporting practices. As such, the journals may be responsible for ensuring the reviewers are adhering to standard reporting practices.

Second, to limit the potential of selective reporting of fit indices, researchers should cite their method of reporting. Multiple methods of reporting fit indices exist. Some methods suggest that researchers report the same indices, such as Kline (2016), who recommends reporting the model chi-square statistic, RMSEA, CFI, and the SRMR. Some researchers suggest one should report the TLI, CFI, and RMSEA for one-time analyses and then only report other fit indices when making modifications to the model (Schreiber et al. 2006). Some suggest a hybrid, where researchers must always report the model chi-square, SRMR and then choose a parsimonious index and a relative index (Ockey and Choi, 2015). Finally, some allow researchers to choose one absolute, incremental, and parsimonious fit index (Jackson et al. 2009). Thus, researchers have many methods to choose from when reporting fit indices.

Still, there are limitations to selecting a method when reporting fit indices. First, every method has limitations. For example, the Kline method does not allow for parsimonious fit indices, which reveal a worse fit for more complex methods. Jackson et al. (2009) method still allows researchers to select the best-fit indices of the domains of fit indices to report. The method of Ockey and Choi (2015) limits researchers to specific indices and allows for the ability to select the fit indices that estimate a better model fit. Moreover, researchers will have the potential to selectively pick a method *post-hoc* that makes their models appear to fit better. This selective use of methods may reduce the selective reporting of fit indices; however, it does not stop them. Therefore, the problem with fit index reporting is not the fit indices themselves; rather, the problem comes from the intention and motivation of the researchers to misrepresent their data-model fit. Thus, researchers still need to work on ways of refining the standardization of reporting of fit indices.

## Using Fit Indices to Justify Poorly Fitting Models

The chi-square exact fit test is sensitive to and suggests poor model fit from minor and typically insignificant model misspecifications (Bentler and Bonett, 1980). With sample sizes between 75 and 200, the chi-square test is typically an appropriate indicator of model fit. However, when the sample size is over 400, most models are rejected. This sensitivity to minor model misspecifications limits the utility of the chi-square exact fit test.

The researcher’s ability to detect if a model fits the observed data is limited due to the chi-square exact fit test sensitivity to sample size, so researchers typically rely on other fit measures. Some researchers may use fit indices to justify poorly-fitted models. For example, we can imagine that researchers are testing a model using a dataset of 400 observations. Almost certainly, the chi-square test will suggest that the model does not fit the data. In this example, imagine that the chi-square test is very high, given the degrees of freedom and sample size (e.g., *χ*^2^(1)=10,000, *p* <0.001). The chi-squared test is much higher than expected, even though the test is sensitive to sample size (i.e., the chi-square test suggests that the model does not fit the data even when accounting for being overpowered). Some fit indices for this model might suggest that the model moderately fits (e.g., a CFI of 0.83). The researcher may then ignore the exact fit test and rely on the CFI to justify the model’s fit. Further, a fit index may appear to suggest good data-model fit even when a majority of the pattern coefficients are nonsignificant or weak. This pattern of reporting may mislead the readers to believe that some models fit the data better than they appear given the chi-square exact fit test and pattern coefficients. The problem is not with the fit indices (i.e., the fit indices report the information they were designed to report); rather, the problem is when researchers use the fit indices to argue that a model fits the data when there are major areas of misfit.

## Future Directions

Researchers should consider the three-step process by Kline (2016) for assessing model fit instead of relying on fit indices. Kline (2016) suggested this method to retain a model as one plausible explanation of the data, even when the exact fit test suggests that the specified model does not fit the data. Step 1 involves fitting the model to the data and reporting the exact fit test. If the model passes the exact fit test, then the researcher will temporarily retain the model as one plausible explanation for the data. If the model fails the exact fit test, then the researcher will tentatively reject the model. Step 2 involves examining standardized and correlational residuals. Standardized residuals are a standardized measure of the error between the observed data and the model-implied data for each piece of unique information in the model-implied covariance matrix. The correlational residuals measure the error between the underlying correlation between items and the model-implied correlations between items. Kline recommends that researchers reject the model if there are numerous correlational residuals (associated with significant standardized residuals) with an absolute value of greater than 0.1 and retain the model if there are no significant correlational residuals. This second step means that researchers may reject models that pass the exact fit test and retain models that fail the exact fit test. Step 3 involves reporting the RMSEA, CFI, and SRMR but not using these fit indices to justify the model fit.

The method of Kline (2016) has several benefits over using the exact fit test or fit indices alone. First, the method of Kline (2016) of examining standardized and correlational residuals allows researchers to assess the fit of individual parts of a model instead of the model as a whole. This benefit allows researchers to assess where the model fits poorly and adjust accordingly (i.e., adding an extra parameter). Second, the method of Kline (2016) allows models that have failed the exact fit test to be redeemed. This benefit removes the emphasis on the exact fit test and allows the researcher to assess if the model failed the exact fit test due to large residuals or just minor model misspecifications. These benefits suggest that using the method of Kline (2016) may be more valid than using fit indices alone.

## Limitations of Fit Indices

Although fit indices provide helpful information in assessing data-model fit, there are several notable limitations. First, simulation studies suggest that the implications of cut-off values change when loading and sample size are manipulated (Sharma et al. 2005). This research suggests that proper cut-offs for fit indices (i.e., 0.95 for CFI; Schreiber et al. 2006) changes as a function of the strength of the loadings from the common factors to the indicators, making these fit index cut-offs unreliable. Second, fit indices measure the average fit of the model across parameters and do not allow researchers to assess for the fit of different parameters. This limitation implies that a model with suitable fitting and poor fitting parameters may give a similar fit index as a model with average fitting parameters across the model. Finally, fit indices are only one of many methods that assist researchers in assessing data-model fit. For example, instead of relying exclusively on fit indices researchers can use relative fit across multiple competing models and select the model that demonstrates the best fit. Further, researchers may consider not relying only on empirical methods to determine a model’s fit, but also instead using theory and logic to determine which models fit better. For example, a model that is weakly justified theoretically but fits the data well (i.e., solely empirically driven) may not be a model of the hypothesized phenomenon that is as valid as a model that does not fit the data as well but has stronger theoretical support (e.g., Box, 1976; Hox and Bechger, 1999). Further, using pluralistic methods over a single method (i.e., fit indices alone), such as the method of Kline (2016), relative fit comparisons, and theoretical justification in addition to fit indices may guard against the misuse of fit indices (Mayrhofer and Hutmacher, 2020; Zitzmann and Loreth, 2021).

## Conclusion

Fit indices in structural equation modeling provide helpful information about the data-model fit; however, researchers should use fit indices responsibly and ethically to assure that they do not misrepresent the fit of models. The suggestions in the current paper may limit the misuse of fit indices; however, researchers may still misuse these suggestions. To maintain the credibility of analyses under the structural equation modeling framework, researchers have a responsibility to uphold the standards of reporting set forth by the experts in the field. The ethical use of fit indices sustains the scientific rigor of the social sciences commanded by empirical investigations. Furthermore, the responsible use of structural equation modeling techniques will allow social scientists to build on the existing framework, which may increase the potential to answer more complex and essential questions.

## Author Contributions

The author confirms being the sole contributor of this work and has approved it for publication.

## Conflict of Interest

The author declares that the research was conducted in the absence of any commercial or financial relationships that could be construed as a potential conflict of interest.

## Publisher’s Note

All claims expressed in this article are solely those of the authors and do not necessarily represent those of their affiliated organizations, or those of the publisher, the editors and the reviewers. Any product that may be evaluated in this article, or claim that may be made by its manufacturer, is not guaranteed or endorsed by the publisher.
